# Teriflunomide promotes oligodendroglial differentiation and myelination

**DOI:** 10.1186/s12974-018-1110-z

**Published:** 2018-03-13

**Authors:** Peter Göttle, Anastasia Manousi, David Kremer, Laura Reiche, Hans-Peter Hartung, Patrick Küry

**Affiliations:** Department of Neurology, Medical Faculty, Heinrich Heine University, Moorenstrasse 5, 40225 Düsseldorf, Germany

**Keywords:** Myelin repair, Multiple sclerosis, Oligodendrocyte, Remyelination, Neuroregeneration, Inhibitor, Transcription factor, p73, p57kip2

## Abstract

**Background:**

Multiple sclerosis (MS) is a neuroinflammatory autoimmune disease of the central nervous system (CNS) which in most cases initially presents with episodes of transient functional deficits (relapsing-remitting MS; RRMS) and eventually develops into a secondary progressive form (SPMS). Aside from neuroimmunological activities, MS is also characterized by neurodegenerative and regenerative processes. The latter involve the restoration of myelin sheaths—electrically insulating structures which are the primary targets of autoimmune attacks. Spontaneous endogenous remyelination takes place even in the adult CNS and is primarily mediated by activation, recruitment, and differentiation of resident oligodendroglial precursor cells (OPCs). However, the overall efficiency of remyelination is limited and further declines with disease duration and progression. From a therapeutic standpoint, it is therefore key to understand how oligodendroglial maturation can be modulated pharmacologically. Teriflunomide has been approved as a first-line treatment for RRMS in the USA and the European Union. As the active metabolite of leflunomide, an established disease-modifying anti-rheumatic drug, it mainly acts via an inhibition of de novo pyrimidine synthesis exerting a cytostatic effect on proliferating B and T cells.

**Methods:**

We investigated teriflunomide-dependent effects on primary rat oligodendroglial homeostasis, proliferation, and differentiation related to cellular processes important for myelin repair hence CNS regeneration in vitro. To this end, several cellular parameters, including specific oligodendroglial maturation markers, in vitro myelination, and p53 family member signaling, were examined by means of gene/protein expression analyses. The rate of myelination was determined using neuron-oligodendrocyte co-cultures.

**Results:**

Low teriflunomide concentrations resulted in cell cycle exit while higher doses led to decreased cell survival. Short-term teriflunomide pulses can efficiently promote oligodendroglial cell differentiation suggesting that young, immature cells could benefit from such stimulation. In vitro myelination can be boosted by means of an early stimulation window with teriflunomide. p73 signaling is functionally involved in promoting OPC differentiation and myelination.

**Conclusion:**

Our findings indicate a critical window of opportunity during which regenerative oligodendroglial activities including myelination of CNS axons can be stimulated by teriflunomide.

## Background

In the past two decades, great progress has been made in the development of immunomodulatory disease-modifying treatments (DMTs) that reduce autoimmune-mediated inflammatory damage in multiple sclerosis (MS). Histopathologically, MS is characterized by an infiltration of immune cells into the central nervous system (CNS) followed by an attack directed against the myelin-producing cells of the CNS, the oligodendrocytes. Their demise results in myelin sheath degeneration, leading to a reduction of axonal conductivity as well as, ultimately, neurodegeneration [1]. The relapsing-remitting form of MS (RRMS) is characterized by periods of immune-mediated axonal demyelination (relapse) which is followed by spontaneous partial remyelination and functional recovery (remission). Resident oligodendroglial precursor cells (OPCs) are dispersed throughout the adult CNS and constitute a reservoir for the generation of new myelin sheath-producing oligodendrocytes even in the aged CNS [2]. Nevertheless, endogenous repair mechanisms are inefficient and often fail to restore function and structure against the backdrop of chronic and/or recurring inflammatory reactions in the CNS [3]. Of note, currently available DMTs only slow down disease progression and decrease the development of new lesions but do not directly promote repair via restoring myelin. Hence, the therapeutic goal to promote long-term repair of existing lesions is still unmet.

Teriflunomide is the active metabolite of the pro-drug leflunomide and is used as an immunomodulatory DMT for RRMS [4]. Its primary mode of action consists in the inhibition of pyrimidine biosynthesis in activated lymphocytes via the selective and reversible blockade of the mitochondrial enzyme dihydroorotate dehydrogenase (DHODH) [5, 6]. This enzyme is central to de novo pyrimidine synthesis, which is rate limiting for the activation and proliferation of lymphocytes [7, 8]. However, pyrimidines within these cells not only serve as a source for DNA/RNA synthesis but are also necessary for protein glycosylation and membrane lipid biosynthesis [9]. Teriflunomide thereby reduces the number of activated peripheral T and B lymphocytes, which could potentially infiltrate the CNS [7]. In contrast, resting lymphocytic cells synthesize pyrimidine through a salvage pathway independent from DHODH and are thus assumed to remain unaffected [7]. Furthermore, teriflunomide was shown to modulate the store-operated calcium entry (SOCE) pathway which is an important source for cytosolic Ca2+ signaling [10]. It is involved in multiple other signaling pathways and cellular processes such as cell cycle regulation, apoptosis, MAPK, and the p53 signaling pathway [11, 12]; the latter of which was implicated in the differentiation of oligodendroglial cells [13–15]. Given that teriflunomide can cross the blood-brain barrier (BBB) with 1–2% of serum concentrations (in the range of 2.5–4.1 μM) reaching the CNS [16–18], we hypothesized that it could exert a direct effect on CNS cells, such as, for instance, a modulation of OPC differentiation and homeostasis. Based on the findings presented here, we can now conclude that short-term teriflunomide stimuli can efficiently promote OPC differentiation resulting in the generation of myelin internodes. We here present evidence that oligodendrogenesis-associated transcription factors, p73 signaling as well as the nuclear export protein chromosome region maintenance 1 (CRM1) are functionally involved in this pro-oligodendroglial process. To our knowledge, this study is the first to describe teriflunomide’s effect on resident CNS cells beyond its general blockade of the mitochondrial enzyme DHODH.

## Methods

### Oligodendroglial cell culture

Generation of primary OPCs from postnatal day zero (P0) cerebral rat cortices (Wistar rats of either sex) was performed as previously described [19]. Anti-A2B5 staining (Merck Millipore, Darmstadt, Germany; cat. no. MAB312R RRID:AB_11213098) revealed that the cultures consisted of 98% oligodendroglial cells (data not shown). OPCs were either kept in proliferation-supporting high-glucose DMEM-based Sato medium (ThermoFisher Scientific, Life Technologies, Darmstadt, Germany), supplemented with 10 ng/ml recombinant human bFGF (PeproTech, Hamburg, Germany) and 10 ng/ml recombinant human PDGF-AA (R&D Systems, Darmstadt, Germany) or differentiation was initiated by Sato medium depleted of growth factors and supplemented with 0.5% fetal calf serum (PAA Laboratories, Cölbe, Germany). Oligodendroglial cells were treated with 0, 1, 5, 10, 12.5, 25, and 50 μM teriflunomide (provided by Sanofi Genzyme, Waltham, USA) in differentiation medium, based on a stock concentration of 100 mM dissolved in dimethyl sulfoxide (DMSO).

### Myelinating co-culture

Dissociated neuron/oligodendrocyte co-cultures were obtained from embryonic day 16 (E16) rat cerebral cortex (Wistar rats of either sex) according to [20] and as previously published by us [21]. Cortical cells were plated on 15-mm poly-D-lysine (0.1 mg/ml) coated cover slips (65,000 cells per cover slip) and kept in myelination medium consisting of N2 and neurobasal medium (ThermoFisher Scientific, Darmstadt, Germany; ratio 1:1) including NGF (50 ng/ml) and NT-3 (10 ng/ml) (both R&D Systems, Minneapolis, USA). The day of primary culture was defined as day 1 in vitro (DIV1). After 10 days in vitro (DIV10), insulin was excluded and the ratio of the insulin-free N2 to neurobasal medium including B27 supplement (ThermoFisher Scientific, Darmstadt, Germany) was adjusted to 4:1. This myelination medium was further supplemented with 60 ng/ml tri-iodo-thyronine (T3, Sigma-Aldrich; Taufkirchen, Germany). Final concentrations of individual N2 medium components (DMEM-F12 based, high glucose; ThermoFisher Scientific, Germany) were insulin (10 μg/ml), transferrin (50 μg/ml), sodium selenite (5.2 ng/ml), hydrocortisone (18 ng/ml), putrescine (16 μg/ml), progesterone (6.3 ng/ml), biotin (10 ng/ml), *N*-acetyl-l-cysteine (5 μg/ml) (all Sigma-Aldrich, Taufkirchen, Germany), bovine serum albumin (0.1%, Roth, Karlsruhe, Germany), and penicillin–streptomycin (50 units/ml, ThermoFisher Scientific, Darmstadt, Germany). At DIV30, cover slips were washed with PBS, fixed with 4% paraformaldehyde, and processed for immunofluorescent staining. At the onset of myelination DIV17, teriflunomide stimulation was either performed from DIV17 until DIV20 (pulse) or until DIV30 (constant stimulation).

### Immunostaining

Fixed cells were permeabilized with PBS containing 0.01% Triton X-100 (Sigma-Aldrich; Taufkirchen, Germany) and unspecific staining was blocked with 10% normal goat serum or donkey serum (Sigma-Aldrich; Taufkirchen, Germany) for 40 min [19]. Cells were then incubated with primary antibodies in PBS overnight at 4 °C using the following dilutions: rat anti-myelin basic protein (MBP; 1/500, Bio Rad., Munich, Germany; cat.no. MCA409S RRID:AB_325004), mouse anti-MBP and anti-2′,3′-cyclic-nucleotide 3′-phosphodiesterase (CNPase) antibodies (1/500 and 1/1000; cat. no. SMI-94R-500 RRID:AB_10124143 and cat. no. SMI-91R-500 RRID:AB_510038, respectively; all Biolegend, CA, USA), rabbit anti-p57kip2 (1/500, Sigma-Aldrich; Taufkirchen, Germany, cat. no. P0357 RRID:AB_260850), rabbit anti-p73 (1/100, Bethyl laboratories, Montgomery, USA), and mouse anti-myelin oligodendrocyte glycoprotein (MOG; 1/500, Merck Millipore, Darmstadt, Germany; cat. no. MAB5680 RRID: RRID:AB_1587278). Fixed co-cultures were blocked with PBS containing 0.5% Triton X-100 and 2% normal goat serum and then incubated overnight in 0.1% Triton and 2% normal goat serum containing primary antibodies anti-MBP (1/250), anti-p57kip2 (1/500), and chicken anti-βIII-tubulin (1/1000, Aves Labs, OR, USA; cat. no. TUJ RRID:AB_2313564). After 24 h, cover slips were washed with PBS and then incubated with secondary antibodies in PBS (diluted 1/500) for 2 h: Alexa Fluor 488 ((ThermoFisher Scientific, Darmstadt, Germany); cat. no. A11001 RRID:AB_10566289; Life Technologies; cat. no. A11034 RRID:AB_10562715), Alexa Fluor 405 (Molecular Probes/(ThermoFisher Scientific, Darmstadt, Germany); cat. no. A31553 RRID:AB_221604), Alexa Fluor 594 ((ThermoFisher Scientific, Darmstadt, Germany); cat. no. A11005 RRID:AB_10561507; Life Technologies; cat. no. A11037 RRID:AB_10561549), and DyLight 405-conjugated antibodies (Rockland Immunochemicals Inc. (Biomol), Hamburg, Germany; cat. no. 603-146-126 RRID:AB_1961602). Nuclei were stained with 4′,6-diamidin-2-phenylindol (DAPI, Roche, Basel, Switzerland). Images (× 20; Zeiss Axionplan2 microscope) were captured using the same light intensity and filters for all images to be compared and processed with Axiovision 4.2 software (Zeiss, Jena, Germany; RRID:SciRes_000111). The analysis was done using Java software (ImageJ, RRID:nif-0000-30,467/Wright Cell Imaging Facility, RRID:nif-0000-30,471). Immunopositive cells were counted in nine randomly chosen fields per coverslip. Two coverslips were used per condition. The total number of cells per field was determined via DAPI staining. For quantification, the number of immunopositive cells was compared to the total cell number and expressed as percentage [mean ± standard error of the mean (SEM)].

### RNA preparation, cDNA synthesis, and quantitative reverse transcription polymerase chain reaction

RNA purification, cDNA synthesis, and determination of gene expression levels by means of quantitative real-time RT-PCR were all performed as previously described [19]. Primer sequences were determined using PrimerExpress 2.0 software (Life Technologies) and tested for the generation of specific amplicons (sequences are available upon request). GAPDH and ODC were used as reference genes, and relative gene expression levels were determined according to the ∆∆Ct method (Life Technologies). Each sample was measured in quadruplicate. Primer used for polymerase chain reaction: CNPase_fwd: ATGCTGAGCTTGGCGAAGAA, CNPase_rev: GTACCCCGTGAAGATGGCC, CRM1_fwd: CACCGCTAAATCCCGGAAGT, CRM1_rev: GGAGATTAGCCACGTAGTCTTGAAT GAPDH_fwd: GAACGGGAAGCTCACTGGC, GAPDH_rev: GCATGTCAGATCCACAACGG, Mash1/Ascl1_fwd: CGT CCT CTC CCG AAC TGA TG, Mash1/Ascl1_rev: TGT AGC CGA AGC CAC TGA AGT, MBP_fwd: CAATGGACCCGACAGGAAAC, MBP_rev: TGGCATCTCCAGCGTGTTC, ODC_fwd: GGT TCC AGA GGC CAA ACA TC, ODC_rev: GTT GCC ACA TTG ACC GTG AC, p53_fwd: GCTTTGAGGTTCGTGTTTGTGCC, p53_rev: AGTCATAAGACAGCAAGGAGAGGGG, TAp73_fwd: AGGGTCTGTCGTGGTACTTTGACC, TAp73_rev: GGTTGTTGCCTTCTACACGGATGAG, p57kip2_fwd: CAGGACGAGAATCAGGAGCTGA, p57kip2_rev: TTGGCGAAGAAGTCGTTCG, rPLP_fwd: CTTTGGAGCGGGTGTGTCAT, rPLP_rev: TGTCGGGATGTCCTAGCCAT, rNkx2.2_fwd: CCTTTCTACGACAGCAGCGA, rNkx2.2_rev: GTCATTGTCCGGTGACTCGT, rSox10_fwd: GCAGGCTGGACACTAAACCC, rSox10_rev: GTGCGAGGCAAAGGTAGACTG, rMyrf_fwd: CCTGTGTCCGTGGTACTGTG, rMyrf_rev: TCACACAGGCGGTAGAAGTG.

### Western blot analysis

For Western blot analysis, six-well plates with 500,000 cells per well were used. On given time points, oligodendroglial cells were detached by means of incubation with pre-warmed trypsin-EDTA (Capricorn Scientific, Ebsdorfergrund, Germany; 37 °C, humidity 98%, 5% CO2, 5 min), before reactions were stopped with culture medium. Cell suspensions were centrifuged (1500 rpm, 10 min, 4 °C), and subsequently cell pellets were subjected to lysis with RIPA buffer (Cell Signaling Technology, Leiden, Netherlands) supplemented with Halt protease and phosphatase inhibitor cocktail (ThermoFisher Scientific Darmstadt, Germany). Whole cell lysates were subjected to two sonication cycles of 15 s each and then centrifuged (14,000 rpm, 10 min, 4 °C) to obtain the soluble protein fraction. Protein concentrations were determined using the DC Protein Assay (BioRad, Munich, Germany). Samples were subjected to standard sodium dodecyl sulfate (SDS) gel electrophoresis and Western blotting using 4–12% RunBlue SDS gels (Expedeon, Cambridgeshire, UK) and RunBlue Blot Sandwich nitrocellulose blotting (E Expedeon, Cambridgeshire, UK), respectively, followed by application of mouse anti-actin (1/2000 cat. no. 612656 RRID:AB_2289199, BD Bioscience, Heidelberg, Germany), rabbit anti-p73 (1/500, Bethyl laboratories, Montgomery, USA), and mouse anti-CNPase (1/300 cat. no. SMI-91R-500 RRID:AB_510038, Biolegend, CA, USA), (MBP; 1/500, Bio Rad., Munich, Germany; cat.no. MCA409S RRID:AB_325004), and (PLP; 1/1000, Abcam, Cambridge, UK; cat.no. ab9311: RRID:AB_2165790) antibodies. For visualization of signals using peroxidase-labeled horse anti-mouse (Vector Laboratories, Burlingame, CA, USA; 1:5000) or HRP-linked goat anti-rabbit (Cell Signaling Technology; 1:2000) secondary antibodies, nitrocellulose membranes were incubated for 5 min with SuperSignal West Pico Chemiluminescent Substrate (ThermoFisher Scientific, Germany). Protein bands were quantified using the Fusion FX software (Vilber Lourmat, Eberhardzell, Germany). The intensity for each band was determined and normalized to the intensity of the actin band of the corresponding probe (ImageJ, RRID:nif-0000-30,467/Wright Cell Imaging Facility, RRID:nif-0000-30,471).

### Statistical analysis

Data are presented as mean ± standard error of the mean (SEM) and significance was assessed by two-sided Student’s *t* test, unpaired comparison for means (GraphPad Prism, RRID:rid_000081). The experimental groups were considered significantly different at *p < 0.05, **p < 0.01, and ***p < 0.001. *n* represents the number of independent experiments.

## Results

Since it was recently demonstrated that teriflunomide exerts direct effects on resident CNS cells such as microglia and astrocytes [18], we hypothesized that teriflunomide could also affect oligodendroglial homeostasis, proliferation, and differentiation all of which are cellular processes important for myelin repair. We therefore investigated the effect of teriflunomide on purified postnatal primary rat OPCs that can mature upon mitogen withdrawal by means of time course stimulation and dose dependency analyses.

### Tolerated dosage of teriflunomide for primary rat oligodendroglial precursors

**Fig. 1:**
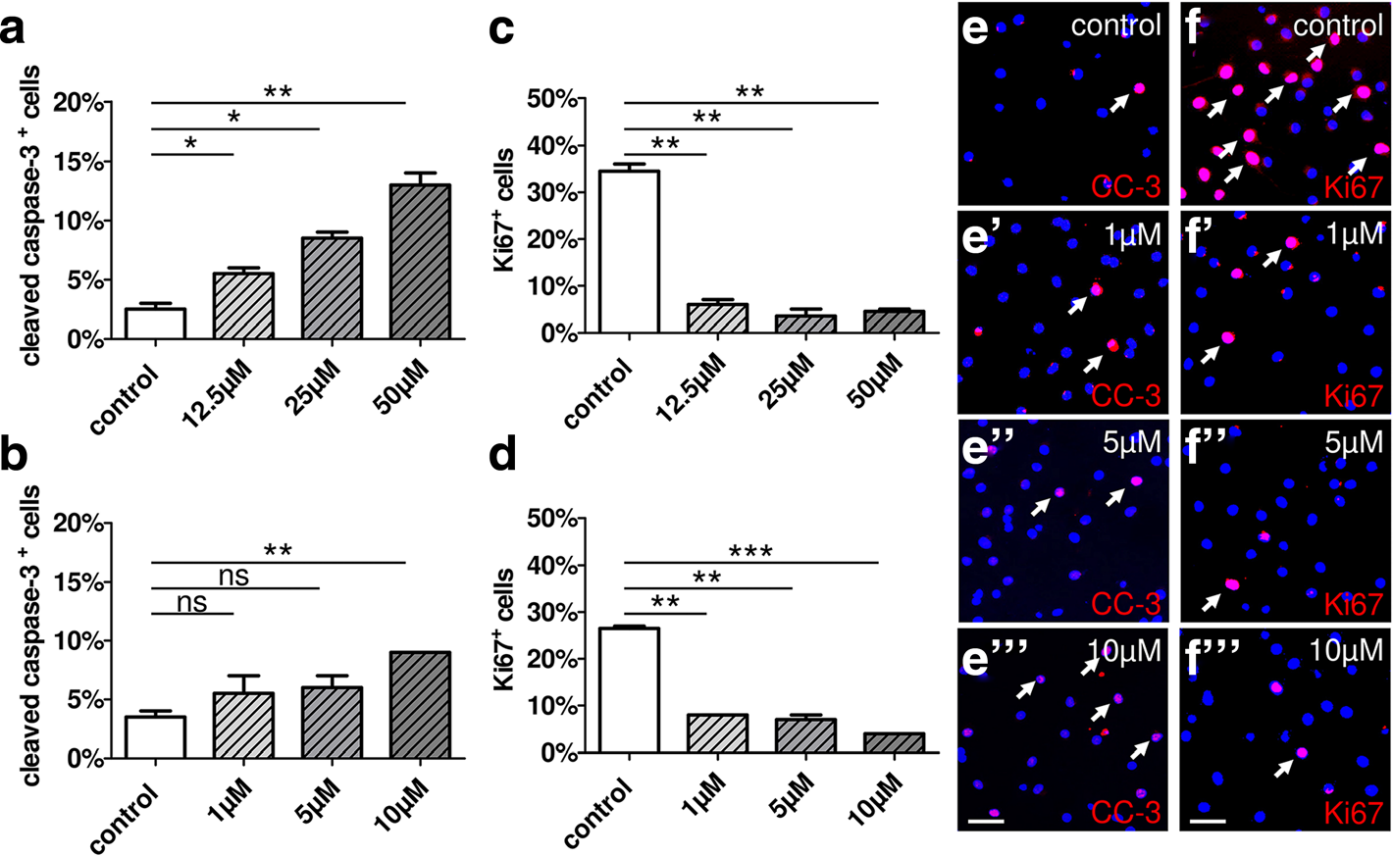
Oligodendroglial cell death and proliferation rates upon stimulation with teriflunomide. **a** Application of high doses of teriflunomide for 1 day (1d) led to a substantial increase in the percentage of apoptotic OPCs cells as judged by the expression of activated caspase-3 (CC-3), whereas low concentration profiles (**b**, **e**–**e”’**) did not significantly change the naturally occurring death rate. Proliferation after 1d was significantly reduced upon high (**c**) and low (**d**, **f**–**f”’**) doses as indicated by the percentage of Ki-67-positive cells. Data are shown as mean; error bars represent SEM. Number of experiments: *n* = 2 for (**a**–**d**). *t* test (ns, not significant, *p < 0.05, **p < 0.01, ***p < 0.001). Scale bars, 30 μM

In order to determine functionally relevant cell-specific concentrations, cell death and proliferation of cultured primary oligodendroglial cells were studied in the presence or absence of teriflunomide using markers such as activated cleaved caspase-3 (CC-3) and the proliferation-associated DNA-binding nuclear protein Ki-67, respectively. To this end, primary OPCs were stimulated with two different concentration series—high (0, 12.5, 25, 50 μM teriflunomide) and low (0, 1, 5, 10 μM teriflunomide)—for 1 day in vitro. Immunofluorescent staining for CC-3 revealed that the high concentration profile substantially increased the degree of apoptotic OPCs whereas the low concentration profile did not (Fig. 1a, b). Furthermore, stimulation of OPCs for 1 day led to a significant reduction of oligodendroglial cell proliferation even at the lowest concentrations of 1 or 5 μM (Fig. 1c, d). These findings suggest that teriflunomide induces pyrimidine stress in oligodendroglial cells ultimately leading to cell cycle exit and impaired cell survival. Therefore, for subsequent stimulation experiments, concentrations of the low range were used.

### Gene expression responses upon teriflunomide stimulation

**Fig. 2:**
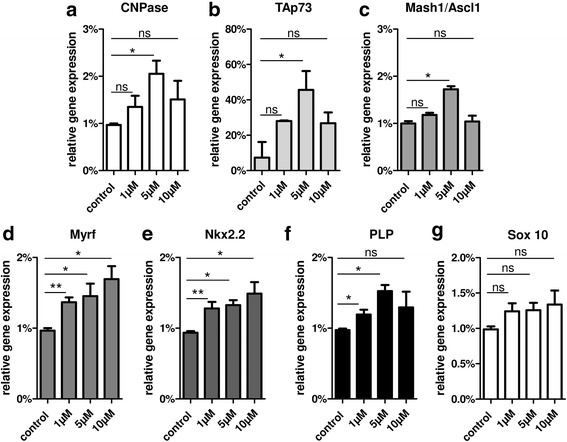
**a**–**g** Gene expression responses upon short-term teriflunomide stimulation. Quantitative RT-PCR after 1d revealed that stimulation of primary rat OPCs with tolerated concentration profiles of teriflunomide led to a significant upregulation of CNPase, TAp73, Mash1/Ascl1, Myrf, Nkx2.2, PLP, and Sox 10 transcript levels. GAPDH was used as reference gene. Data are shown as mean, and error bars represent SEM. Number of experiments: *n* = 3. *t* test (ns, not significant, *p < 0.05, **p < 0.01)

To further investigate specific reactions, primary OPCs were then stimulated with 1, 5, and 10 μM teriflunomide for 24 h and subsequently transcript levels of differentiation-associated markers were determined by means of quantitative real-time PCR (qRT-PCR). Of note, a concentration of 5 μM appeared to be promising in that transcript levels of myelin markers such as 2′,3′-cyclic-nucleotide 3′-phosphodiesterase (CNPase) and myelin proteolipid protein (PLP) were upregulated at 1 day (Fig. 2) and at 3 days (CNPase, data not shown; PLP, Fig. 6). This reaction was furthermore accompanied by a change in the expression of key oligodendroglial transcription regulators required for proper oligodendroglial cell differentiation and myelin induction such as Mash1/Ascl1 [22], myelin regulatory factor Myrf [23], the homeodomain transcription factor Nkx2.2 [24, 25] and TAp73 [14], whereas a non-significant increase in Sox10 expression was observed (Fig. 2). TAp73 represents a splice variant of the p73 gene generated from P1 promoters containing a complete N-terminal transactivation domain (TA) that is transcriptionally proficient compared to ∆N isoforms lacking the TA rendering them transcriptionally inactive [26].

### Oligodendroglial maturation marker and regulator expression upon pulsed teriflunomide application

**Fig. 3:**
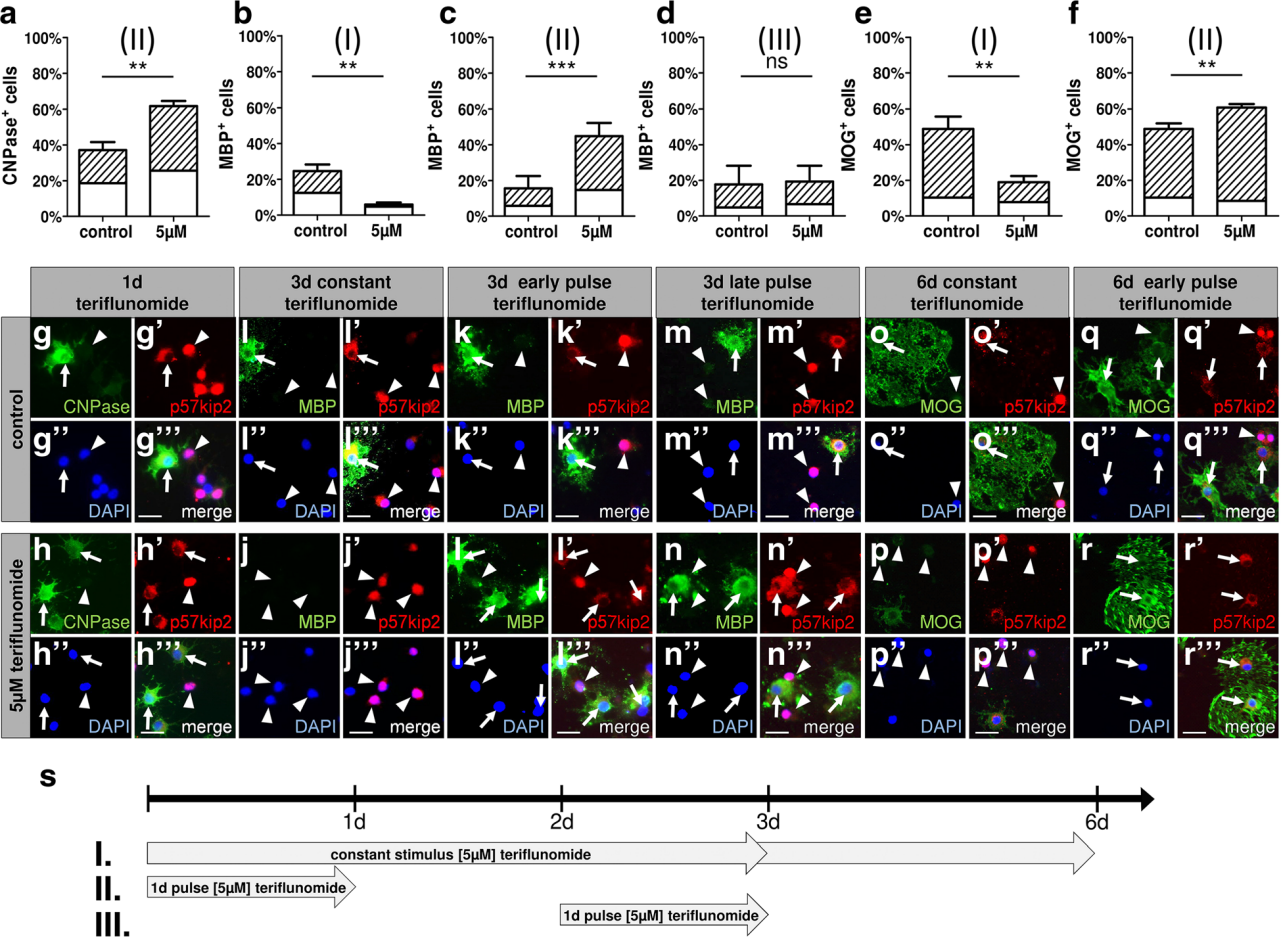
OPC differentiation dynamics and marker protein responses as a function of different teriflunomide application schemes. **a**–**f** Percentage of oligodendroglial cells positive for myelin markers displaying nuclear (white bars) or cytoplasmic (dashed bars) p57kip2 signals. **a**, **g**–**h”’** After short-term stimulation with 5 μM teriflunomide (**s**; scheme II), an increase of CNPase-positive cells was observed along with increased translocation of p57kip2 from the nucleus (arrowheads) to the cytoplasm (arrows). **c**, **k**–**l”’** A 24 h pulse stimulation (scheme II) with teriflunomide followed by a 48-h (or 120-h; **f**, **q**–**r”’**) withdrawal period led to a substantial increase in the fraction of oligodendroglial cells that translocated p57kip2 from the nucleus to the cytoplasm and correlated with an increase of MBP- and MOG-positive cells, respectively. **b**, **i**–**j”’** On the other hand, long-term stimulation (scheme I) over 72 h (or 144 h; **e**, **o**–**p”’**) resulted in a decrease of cells expressing MBP or MOG and boosted nuclear accumulation of the p57kip2 protein. **d**, **m**–**n”’** Moreover, a short-term pulse at a later time point from 48 to 72 h (scheme III) did not affect oligodendroglial differentiation. Data are shown as mean, and error bars represent SEM. Number of experiments: *n* = 3 (**a**–**f**). *t* test (ns, not significant, **p < 0.01, ***p < 0.001). Scale bars, 20 μM

In order to confirm a presumptive pro-oligodendrogenic role, we focussed on early time points and lower teriflunomide concentrations. In a subsequent series of experiments, a comprehensive set of markers (precursor-, competence-, myelin-, and late maturation markers) was screened in order to determine differentiation-related protein expression levels. Most notably, this investigation revealed that oligodendroglial differentiation responses to teriflunomide were limited to short stimulation periods (Fig. 3). We observed an induction of CNPase protein expression after 24 h of stimulation with 5 μM teriflunomide (Fig. 3a, g–h”’; scheme II) whereas a continued stimulation for 72 h decreased the number of cells expressing CNPase (data not shown) as well as of cells expressing the intermediate myelin maturation marker myelin basic protein MBP (Fig. 3b, i–j”’; scheme I). Moreover, further continued stimulation up to 144 h also impacted the expression of the late myelin maturation marker myelin oligodendrocyte glycoprotein MOG (Fig. 3e, o–p”’). This indicates that stage-, period-, or duration-related secondary signaling pathways are likely to be activated limiting initial pro-oligodendrogenic effects. This hypothesis is supported by the observation that a 24-h teriflunomide pulse followed by a 48-h (for MBP) or 120-h (for MOG) withdrawal period led to an OPC population with a substantially increased fraction of MBP-positive cells (Fig. 3c, k–l”’; scheme II) as well as of MOG-positive cells (Fig. 3f, q–r”’; scheme II). On the other hand, a short pulse at a later time point (from 48 to 72 h) did not result in any differentiation-associated effect (Fig. 3d, m–n”’; scheme III), indicating that early, immature OPCs are most sensitive to teriflunomide-mediated pro-oligodendroglial signals.

The observation that only a transient teriflunomide application promotes oligodendroglial differentiation is further supported by p57kip2 protein localization data. As previously demonstrated by our research group, translocation of this regulatory protein from OPC nuclei to the cytoplasm is essential for their differentiation competence [21]. We therefore examined if teriflunomide stimulation altered the number of cells with cytoplasmic p57kip2 signals. Short-term treatment (24 h only or 24 h pulse with subsequent 48 or 120 h withdrawal; scheme II) increased the percentage of differentiation competent cells (see dashed areas in bars of Fig. 3a, c, f) whereas constant/or late pulse treatments (schemes I and III) significantly reduced the percentage of p57kip2-translocated cells (dashed bars in Fig. 3b, d, e).

**Fig. 4:**
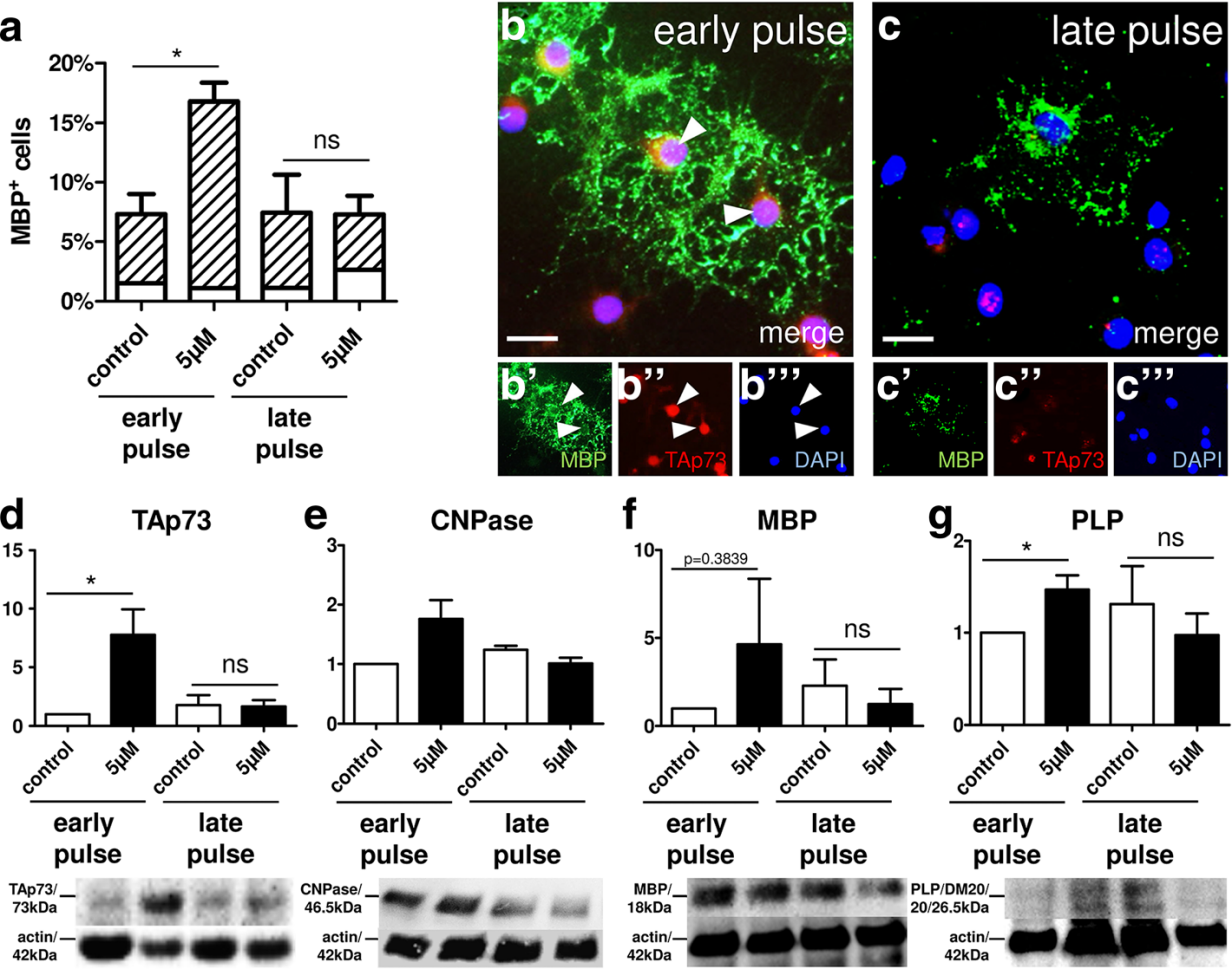
p73 protein induction in response to teriflunomide stimulation. **a**–**c”’** Double immunostaining and its quantification confirmed that early teriflunomide pulses (scheme II, see Fig. 3) result in cells displaying strong TAp73 signals (arrowheads in **b**) correlating also with MBP positivity (dashed bars in **a**). Late teriflunomide pulses (scheme III) could not boost both protein markers. Scale bars, 20 μm. **d**–**g** Western blot analysis confirmed that early short-term teriflunomide pulses (scheme II) result in a strong induction of CNPase (**e**), MBP (**f**), and PLP levels (**g**), as well as of TAp73 (**d**) protein levels. Late pulses (scheme III) did not upregulate TAp73 or myelin marker expression (as quantified for TAp73 in **d**). Actin was used for normalization and protein molecular weights are indicated in kilodalton. Data are shown as mean, and error bars represent SEM. *t* test (ns, not significant, *p < 0.05). Number of experiments: *n* = 3 for (**a**, **d**, **f**, **g**), *n* = 2 for (**e**)

Based on the previously reported differentiation associated role for p73 in oligodendroglial cells [14], we investigated to what degree differentiation promoting vs. differentiation non-promoting stimulation schemes (II vs. III) affected its expression in primary rat OPCs. Double immunostaining of OPCs after 3 days in culture confirmed that upon an early teriflunomide pulse (scheme II), TAp73-positive cells (arrowheads) were also MBP-positive (Fig. 4b–b”’) whereas a late pulse (scheme III) did not affect TAp73 expression and did not induce MBP expression (Fig. 4c–c”’). Further quantification revealed that an early pulse (scheme II) resulted in an increase of strong TAp73 expressing oligodendroglial cells (Fig. 4a; dashed bars). Of note, strong TAp73 expression significantly correlated with the accumulation of MBP in contrast to low TAp73-expressing cells (Fig. 4a; white bars). Western blot analysis confirmed that an early teriflunomide pulse (from 0 to 24 h; scheme II) resulted in a strong induction of TAp73 protein levels accompanied by elevated CNPase, MBP, and PLP protein expression levels whereas a late pulse (from 48 to 72 h; scheme III) had only a minor effect on protein expression levels (quantified in Fig. 4d–g).

### In vitro myelination upon pulsed teriflunomide application

**Fig. 5:**
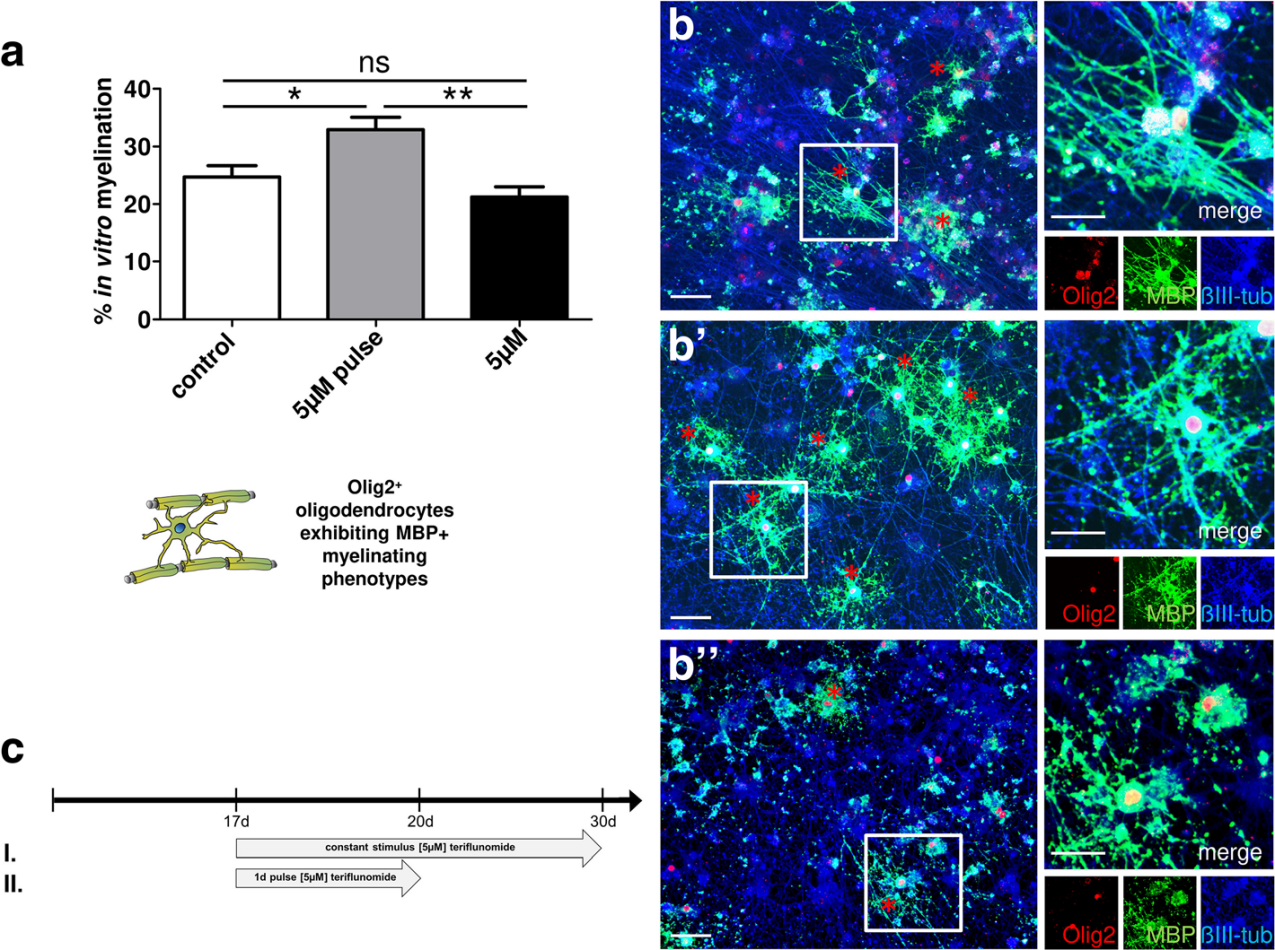
Teriflunomide-dependent modulation of myelination in vitro*.* Myelinating neuron/oligodendrocyte co-cultures were fixed and stained after 30 days in vitro (DIV30). **a** Early short-term (3d) teriflunomide pulse stimulation (scheme II; gray bar; **b’**) significantly increased the number of Olig2-positive oligodendrocytes that formed MBP-positive internodes (asterisk) as compared to controls (white bar; **b**) or long-term stimulation (scheme I; black bar; **b”**). **b**–**b”** Representative triple staining for Olig2, MBP, and ßIII-tubulin. Scale bars, 30 μm. Data are shown as mean, and error bars represent SEM. *t* test (ns, not significant, *p < 0.05, **p < 0.01). Number of experiments: *n* = 5 for (**a**)

Following the observation that early teriflunomide pulses can promote OPC differentiation in monocultures, we aimed at translating our results to myelinating neuron/glia co-cultures (Fig. 5) as established and previously demonstrated in [21]. Mixed rat neuron/glia cultures were grown for 17 days and then treated for another 13 days with myelination medium in order to observe axonal wrapping and the establishment of myelin sheaths (internodes). Due to the complexity of these experiments as well as to their long-term duration, only a comparison of early pulse vs. long-term stimulation was conducted (schemes II vs. I). Teriflunomide (5 μM in myelination medium) was either added immediately after the 17-day initiation period for 3 days (72-h pulse; scheme II) followed by another 10 days of culture in myelination medium only or was added for 13 days at a concentration of 5 μM in myelination medium (constant stimulation; scheme I). After a total duration of 30 days in culture, the percentage of MBP-positive oligodendrocytes exhibiting a myelinating phenotype (i.e., featuring internodes) was determined based on the total number of Olig2-positive cells. This analysis revealed that an early teriflunomide pulse significantly increased the generation of myelinating oligodendrocytes as compared to controls whereas a long-term stimulation did not affect this final maturation process (Fig. 5).

### Differentiation-associated gene expression upon pulsed teriflunomide application

**Fig. 6:**
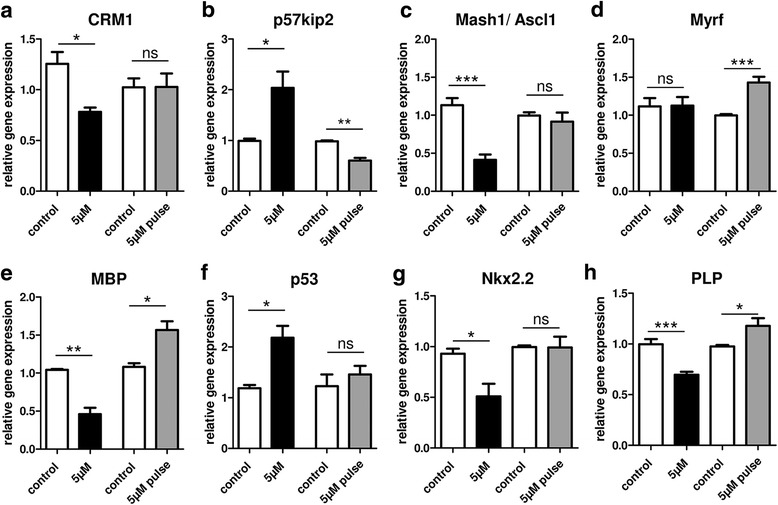
Differential gene expression responses upon constant versus pulsed teriflunomide stimulation. Quantitative RT-PCR upon constant teriflunomide stimulation (scheme I, Fig. 3; black bars) revealed a significant decrease in transcript levels of CRM1 (**a**), MBP (**e**), PLP (**h**), and of transcription factors Mash1/Ascl1 (**c**) and Nkx2.2 (**g**), whereas expression of the oligodendroglial differentiation inhibitor p57kip2 (**b**) as well as of the stress response protein p53 (**f**) were increased. Short-term teriflunomide pulse stimulation (scheme II; gray bars) exerted no significant impact on the expression of CRM1, Mash1/Ascl1, Nkx2.2, and p53 but significantly reduced the expression of the OPC differentiation inhibitor p57kip2 and induced Myrf (**d**), MBP, and PLP transcript levels. GAPDH and ODC were used as reference genes. Data are shown as mean, and error bars represent SEM. *t* test (ns, not significant, *p < 0.05, **p < 0.01, ***p < 0.001). Number of experiments: *n* = 3 for (**a**–**h**)

Finally, investigating the underlying mode of action leading to different oligodendroglial reactions dependent on different stimulation schemes and periods, transcript levels of a number of differentiation-associated genes were analyzed (Fig. 6). Analysis of the 72-h time point revealed that constant teriflunomide stimulation (scheme I) decreased chromosome region maintenance 1 (CRM1; exportin 1) transcript levels whereas an early teriflunomide pulse (scheme II) did not affect CRM1 expression. The CRM1 protein is necessary for the nuclear export of proteins through the nuclear pore complex (NPC), and its transcript levels were lowered in parallel to boosted p53 expression levels. This observation is in line with previous studies demonstrating that CRM1 transcription is inhibited by elevated levels of p53, as this gene was shown to repress CRM1 promoter activity in response to DNA damage [27]. This suggests a complex regulatory loop, whereby low CRM1 levels lead to a decreased protein export into the cytoplasm. Of note, according to our previous findings, this CRM1-mediated process is necessary for nuclear export of the inhibitory p57kip2 protein, which in turn is a prerequisite for OPC differentiation [21]. p57kip2 protein nuclear export was indeed induced by pulsed, early teriflunomide application (Fig. 3a, c; scheme II) whereas constant application abolished cytoplasmic localization of this protein (Fig. 3b; scheme I). In accordance with this hypothesis, the expression of p57kip2 was found to be elevated upon constant teriflunomide application but significantly lowered upon the differentiation promoting teriflunomide early pulse. Furthermore, the expression of Mash1/Ascl1, a transcription factor required for proper oligodendroglial differentiation [22] and functional binding partner of the p57kip2 protein [21] as well as Nkx2.2, a transcription factor allowing induction of myelin gene expression [25, 28] were also decreased upon constant stimulation, in parallel to lowered MBP and PLP transcript levels. In contrast, early teriflunomide pulse (scheme II) resulted in a stabilization of Nkx 2.2 and an induction of Myrf, one of the central regulators of the myelination process [23] as well as of MBP and PLP.

## Discussion

While there are many highly effective treatments reducing inflammatory activity and relapse rate in MS, therapies that promote myelin repair are still unavailable. Tapping into the regenerative potential of cell reservoirs such as OPCs or adult neural stem cells (NSCs) therefore represents one of the biggest currently unmet clinical needs [29]. In order to achieve remyelination, two major approaches are conceivable: The achievement of either an exogenous neutralization of inhibitory elements or the implementation of a direct stimulation of regenerative mechanisms [30]. To this end, new drugs will have to be developed or already approved drugs with regenerative properties will have to be repurposed. The past 10 years have seen tremendous progress in both fields as evidenced by the clinical trials evaluating the anti-LINGO1 antibody opicinumab [31] or the antihistamine/anticholinergic drug clemastine [32] for efficacy in MS and optic neuritis (ON), respectively. However, with the opicinumab trial not meeting its primary endpoint [31] and clemastine only achieving limited effects, the search for regeneration-conferring substances is ongoing [29].

In the present study, we demonstrate that teriflunomide induces transcriptional regulators required for proper oligodendroglial cell differentiation such as Mash1/Ascl1, Sox 10, Myrf [23], Nkx2.2 [24, 25], and TAp73 and increases myelin expression when OPCs are exposed to timed pulses of the substance early during spontaneous differentiation. This observation was corroborated by a change in the subcellular localization of p57kip2 in stimulated cells, which we had previously identified as a regulator of oligodendroglial differentiation competence [21, 33]. Of note, such pro-oligodendroglial teriflunomide concentrations did not significantly reduce cell survival as opposed to higher concentrations. We confirmed our results in myelinating neuron/glia co-cultures demonstrating that early teriflunomide pulses also increase internode formation by oligodendroglial cells. Of note, in this more complex culture paradigm, an extended (constant) teriflunomide application did not decrease myelination indicating that prolonged application as occurring in the context of the RRMS therapy is most probably not counterproductive to repair. Mechanistically, we could demonstrate that long-term application of teriflunomide for 3 days led to a downregulation of CRM1 and Nkx2.2, the latter of which was shown to cooperate with Mash1/Ascl1 [28], and direct binding to corresponding regulatory regions was demonstrated to result in activation of myelin related genes such as ceramide galactosyltransferase [34, 35], PLP [24], and MBP [36]. In addition, experiments in *Nkx2.2*-null mutant mice revealed that the differentiation of MBP- and PLP/DM20-positive oligodendrocytes is dramatically retarded [25], corresponding to our gene expression (Figs. 2 and 6) and protein expression data (Fig. 4). On the other hand, downregulation of CRM1, which is relevant for the shuttling of p57kip2 protein from the nucleus into the cytoplasm [21], hence also affects the activity of the pro-oligodendroglial transcription factor Mash1/Ascl1. Of note, early teriflunomide pulses significantly boosted Myrf transcript levels, a transcription factor essential for myelination during development but also shown to contribute significantly to myelin repair [37]. Moreover, the transcription factor TAp73 was found to be upregulated by teriflunomide stimulation specifically in immature OPCs while mature cells appeared to be unaffected. Whether this is due to a lowered sensitivity of matured cells towards teriflunomide or whether additional signaling cascades are initiated later on which neutralize or counteract pro-oligodendroglial subcellular processes remains to be shown by future studies. TAp73 belongs to the p53 superfamily of transcription factors, which, despite their strong homology, have acquired a high degree of functional specificity. TAp73 seems to be highly relevant in neurogenesis as respective knock-out mice feature, among other abnormalities, hippocampal dysgenesis and hydrocephalus [38]. Moreover, increased tumorigenesis as demonstrated in p53 knock-out animals is not observed [38]. Furthermore, teriflunomide was shown to have an inhibitory impact on store-operated Ca^2+^ influx (SOCE)-mediated calcium signaling [10] which is relevant for OPC proliferation [39, 40] explaining the observed reduction of Ki67-positive OPCs (Fig. 1).

Of note, teriflunomide concentrations used in our experiments were matched to the concentrations reaching the brain during orally administered therapy (2.5–4.1 μM) at least during inflammatory relapses where a collapse of the blood-brain barrier (BBB) can be observed [16, 17, 41]. While this study demonstrated that beyond its well-described effect on inflammation via targeting the proliferation of activated lymphocytes, teriflunomide can also modulate OPC differentiation and myelination; it also showed that there is a specific window of opportunity for such modulation. In how far these effects can be harnessed to contribute to neurorepair in MS, potentially in the context of transiently elevated teriflunomide levels in response to BBB impairment, remains to be elucidated. Finally, pulsed teriflunomide applications might be feasible in patients under other neuroimmunological treatments and may thus provide an add-on effect on tissue restoration.

Furthermore, short-term teriflunomide treatment could also be beneficial in other CNS diseases with white matter damage such as amyotrophic lateral sclerosis (ALS), multiple system atrophy (MSA), or Alzheimer’s disease (AD; [30]) when applied during specific disease stages. Future studies in inflammatory and non-inflammatory CNS disease models must therefore be conducted using different application schemes in order to clarify teriflunomide’s potential as a regenerative compound for biomedical translation. In this regard, it will also be highly relevant to confirm that an extended teriflunomide application cannot harm basic myelination levels in vivo.

## Conclusion

This study describes a new effect of teriflunomide beyond its immunomodulatory role as we could show that under defined circumstances, this medication also promotes oligodendroglial differentiation, which constitutes a prerequisite for myelin repair. It remains to be shown, however, to what extent and with which application schemes endogenous remyelination can be promoted in vivo. Herein certainly lies one of the limitations of this study as we analyzed the impact of teriflunomide on rodent cells in a purely in vitro approach. Thus, in order to provide more conclusive evidence that teriflunomide could, indeed, support remyelination in MS, our results will have to be replicated in in vivo animal demyelination models. In this regard, it will, moreover, be necessary to translate our data to human cells. Hopefully, these future experiments will shed more light on the question in how far long-term teriflunomide application in the context of RRMS therapy can positively impact neurorepair.

## Abbreviations

BBB: Blood-brain barrier
CC-3: Cleaved caspase-3
CNPase: 2′,3′-Cyclic-nucleotide 3′-phosphodiesterase
CNS: Central nervous system
CRM1: Chromosome region maintenance 1
DAPI: 4′,6-Diamidin-2-phenylindol
DHODH: Dihydroorotate dehydrogenase
DMTs: Disease-modifying treatments
MBP: Myelin basic protein
MOG: Myelin oligodendrocyte glycoprotein
MS: Multiple sclerosis
Myrf: Myelin regulatory factor
*n*: Number of experiments
NPC: Nuclear pore complex
NSCs: Neural stem cells
ON: Optic neuritis
OPC: Oligodendroglial precursor cell
P0: Postnatal day zero
PLP: Proteolipid protein
qRT-PCR: Quantitative real-time PCR
RRMS: Relapsing-remitting form of MS
SDS: Sodium dodecyl sulfate
SEM: Standard error of the mean
SOCE: Store-operated calcium entry
SPMS: Secondary progressive form of MS
TA: Transactivation domain
